# TLS-Detectable Plane Changes for Deformation Monitoring

**DOI:** 10.3390/s22124493

**Published:** 2022-06-14

**Authors:** Klemen Kregar, Aleš Marjetič, Simona Savšek

**Affiliations:** Faculty of Civil and Geodetic Engineering, University of Ljubljana, Jamova 2, 1000 Ljubljana, Slovenia; ales.marjetic@fgg.uni-lj.si (A.M.); simona.savsek@fgg.uni-lj.si (S.S.)

**Keywords:** deformations, detectable plane changes, TLS, robotic total station, wooden board platform, tilting, shifting, χ^2^ statistical testing

## Abstract

TLS is nowadays often used for deformation monitoring. As it is not able to scan identical points in different time epochs, mathematical models of objects derived from point clouds have to be used. The most common geometric form to describe built objects is a plane, which can be described by four parameters. In this study, we aimed to find out how small changes in the parameters of the plane can be detected by TLS. We aimed to eliminate all possible factors that influence the scanning. Then, we shifted and tilted a finite physical representation of a plane in a controlled way. After each controlled change, the board was scanned several times and the parameters of the plane were calculated. We used two different types of scanning devices and compared their performance. The changes in the plane parameters were compared with the actual change values and statistically tested. The results show that TLS detects shifts in the millimetre range and tilts of 150″ (for a 1 m plane). A robotic total station can achieve twice the precision of TLS despite lower density and slower performance. For deformation monitoring, we strongly recommend repeating each scan several times (i) to check for gross errors and (ii) to obtain a realistic precision estimate.

## 1. Introduction

### 1.1. Use of TLS for Deformation Monitoring

Temporary and permanent deformations occur in engineering structures due to natural and artificial forces. Our goal was to identify these deformations. In engineering geodesy, especially in the field of deformation and displacement measurements of structures, traditional surveying methods are usually used. Heights and vertical displacements are measured with high-precision levelling methods [1], while spatial displacements and movements are derived from total stations and theodolites [2]. Determining the shapes and positional changes of civil engineering objects is one of the main applications of engineering geodesy.

Terrestrial laser scanning (TLS) is one of the most effective technologies for data acquisition in cases where 3D information with a high resolution is required. Laser scanners have, thus far, not been frequently used for high-precision engineering geodesy measurement tasks, such as deformation monitoring, since the technical specifications of most laser scanners do not fulfil the accuracy requirements in such applications [3]. TLS allows one to acquire the geometries of objects and surfaces with high spatial resolution and high accuracy over large areas, making TLS a potentially attractive technology for structural monitoring and geomonitoring [4]. For these purposes, a sequence of scans must be acquired at different epochs and changes between the epochs need to be detected and quantified by analysing the resulting point clouds. Point-to-point comparisons between two successive measurement times (epochs) cannot be performed with TLS due to the technique’s inability to aim at a specific point. The advantage of this technique lies in the large sets of points captured within a short period of time. However, these points need to be processed properly to yield accurate results [5]. An essential step in this analysis involves the transformation of the point clouds into a common, stable reference system [6]. To solve the problem of a stable reference system and assure the high quality of possible position changes of the point clouds, scanning is usually integrated with two complementary surveying techniques: high quality static GNSS positioning and precise tacheometry [7].

The problem of non-identical points can be solved by adjusting simple geometric models based on the point clouds. Most of the constructed objects can be described or divided into smaller mathematically determined shapes such as planes, spheres, and spline surfaces.

In the following section, we discuss how small plane changes, in terms of translation or rotation (declination) of the plane, can be detected by TLS.

### 1.2. Motivation

Geodetic monitoring was performed on the water barrier at a hydro power plant. The task was to detect the deformations of the object. The concrete flat-shaped wall was scanned in two time epochs. The point cloud was segmented to smaller areas. For each of them, the changes of plane parameters were investigated. However, what changes are permissible and when are they statistically significant? In this study, we focused on the actual ability of the terrestrial laser scanner to detect such changes. Therefore, the position changes of plane primitive represented physically by the board plane were investigated. We compared an actual terrestrial laser scanner (Riegl VZ-400) with a robotic total station (Leica MS50) featuring a scanning function.

In a previous paper [8], we presented a methodology to evaluate overall changes in the plane parameters between successive measurement epochs. Theoretically, the detection rate depends on the precision of the parameters. Since the precision is derived from redundant observations using least-squares adjustments, it is often overestimated when dealing with large point clouds. The main scientific question, therefore, remains as follows: What are the threshold values of the plane-parameter changes that can be empirically detected with the TLS method?

This question will be systematically examined in the following sequel. In this article, we highlight the developments in TLS systems that also allow the use of such systems in deformation monitoring. For a reliable analysis, it is important to know the displacement and inclination of the board and that the plane parameters are calculated from several iterations of the scan of the board in each position. This type of research is important because it provides additional information about the usability and reliability of the TLS system for studying deformations.

### 1.3. Literature Overview

Generally, deformations describe either geometrical changes in shape and dimension (i.e., relative movements inside the object’s surface) or rigid body movements [9] (p. 705), [10]. Due to its measurement accuracy, high point density, and measurement speed, laser scanning increasingly represents an alternative to and/or an additional option for geodetic and photogrammetric data acquisition methods [11]. Various factors, such as point cloud processing methods, scan distance and angle, point cloud density, and number and frequency of iterations, influence the comparison of point clouds. The better we can estimate the influential variables, the more realistically we can analyse point clouds and estimate the behaviour of the object or structure under consideration [12]. With TLS, we can monitor the behaviour of a massive concrete structure. By segmenting and correctly pre-processing the point clouds of individual scans, we can determine the local movements of individual segments in addition to the movements of the entire structure [13].

The development of terrestrial laser scanners has substantially increased the importance of areal measurements in engineering geodesy. Among these, areal deformation measurement (ADM) techniques provide the opportunity to extend the observed region to a large portion of a structure instead of merely measuring a set containing a few discrete control points [14]. Previously, Harmening and Neuner [15] published a research project that aimed to develop a spatiotemporal continuous collocation for describing areal deformations.

TLS data have been used for various engineering applications, such as building information modelling (BIM) [16], surveying and monitoring technical infrastructure [17], and monitoring structural deformations [18,19]. Many more studies need to be performed to enable researchers to monitor deformations at a millimetric magnitude, e.g., in the fields of landslides and rockfalls [20,21], large concrete dams [22], bridges [23], and viaducts [24]. Researchers previously determined the usefulness of TLS in determining subsidence and detecting irregularities in the geometry of railway tracks [25]. Some researchers have also tested other sensors for monitoring deformations in dams, such as non-contact interferometric radar sensors [26], and have obtained positive results compared to the measurements obtained using coordinatometers. The monitoring of wind power plants is mainly accomplished with sensor systems attached to the object, such as inclinometers and accelerometers. Hesse et al. [27] conducted a deformation analysis of a wind energy turbine by investigating the amplitude spectrum of a time series from laser-scanning data. Schill and Eichhorn [28] tested the possibility of contactless monitoring for wind power plants using TLS. Pesci et al. [29] proposed a data analysis strategy in which TLS-based morphological maps computed as point-to-primitive differences are created to monitor damaged buildings during earthquakes.

To ensure the metrical integrity of the laser scanner data for deformation analysis, it is important to independently evaluate the scanner’s performance within a calibration scheme. Tsakiri et al. [30] presented a review of calibration methods for laser scanners, considering both independent procedures and self-calibration measures depending on the scanner system. Terrestrial laser scanners suffer from internal misalignments, leading to systematic measurement errors. Hence, it is necessary to account for systematic errors within the deformation analysis to obtain unbiased results. Holst et al. [31] presented and compared several strategies for dealing with these TLS misalignments without the need for a previous calibration. To guarantee the quality of measurements and obtain realistic deformation and analysis results, it is essential to be aware of all error sources and their impacts on the measurements. A synthetic covariance matrix for terrestrial laser scanning is necessary to model the impacts of the main error sources on the variances, covariances, and correlations within point clouds [32].

An estimated deformation field may be caused by errors in the point cloud, including general point cloud errors, registration errors, modelling errors, and cloud-comparison errors. Therefore, a deformation monitorable indicator (DMI) must be determined to distinguish deformation from errors in the point cloud. DMI also describes the minimum deformation that can be extracted from the point cloud to assess the capability of deformation monitoring [33].

Due to the limited accuracy of scanners, the possibility of using TLS in deformation monitoring remains uncertain. To increase the accuracy of the results, the chosen parts of a monitored structure can be approximated by single geometric entities (small planar surfaces) using orthogonal regression. In this case, the position of a measured point (part of structure) is calculated from scanned points instead of a single measurement [34]. Researchers use different approaches to identify changes between two scans. The most widely adopted approach is to measure changes along the local surface normal, which can become less relevant as the movement direction deviates from the surface normal. Williams et al. [35] provided a supplementary tool for cloud-to-cloud comparisons, for which the choice of a tool should be made based on the expected dominant movement direction (DMD) deviation from the surface normal. For point cloud comparisons, some researchers have proposed considering the errors in point coordinates. This methodology is based on computing the distances between the points of two point clouds in the normal direction of one of the point clouds and repeating the calculations by resampling the point clouds. This process can determine the simulation intervals for the distances between both point clouds, instead of providing only a single value for the distance. Consequently, it is possible to determine, at a specific simulation level, if the differences between both point clouds are significant [36]. By scanning a paraboloid surface with a terrestrial laser scanner and determining the focal length variations and local deformations from best-fit approximations, Holst et al. [37] analysed how the reflectors of radio telescopes deform due to gravitation when changing their elevation angles.

Recently, researchers have explored the applicability of using TLS in a motor-vehicle-based mobile mapping system with a high degree of automation for cost-efficient deformation monitoring of anchored retaining structures along public roads [38]. Scaioni and Wang [39] published a review of modern techniques that were recently applied to dam-deformation measurements.

Although individual sample points are low in precision and, therefore, preclude their use in deformation monitoring, modelling the entire point cloud may be effective for representing changes in the shape of a structure [40].

## 2. Methodology

### 2.1. Parameters and Precision Estimation

**Figure 1:**
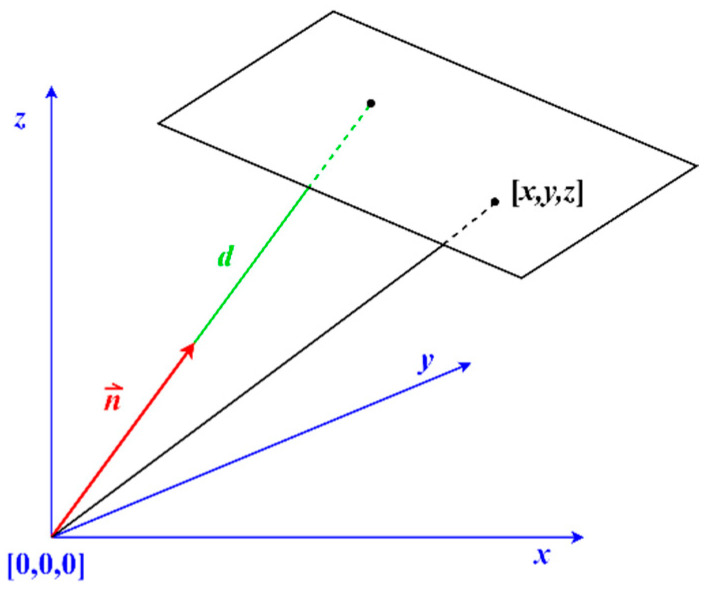
Mathematical model of the plane.

For each scan of the board plane, the parameters were extracted. The mathematical model of the plane is as follows:

(1) ax+by+cz+d=0,

where a, b, and c are components of the unit vector of the plane’s normal and d is the distance of the plane from the coordinate origin in the direction of the normal. Figure 1 graphically illustrates the mathematical model of the plane (Equation (1)).

For large datasets (i.e., those with $10^{5}$ measured points on the plane), the most efficient way to compute the plane parameters is to look for the eigenvectors of the covariance matrix of the measured points [41]. Let X be a matrix of size n×3 including the 3D coordinates of all n points scanned on the plane. Then, the covariance matrix $\Sigma_{X}\;\left(3\times 3\right)$ represents the dispersion of points in $R^{3}$, and the eigenvector belonging to the smallest eigenvalue represents the normal of the plane since the dispersion of points is the smallest in the direction of the normal.

Before calculating the parameters, a constant value of coordinates near the centre of the board is subtracted from all point cloud coordinates. The coordinate system origin should be located near the plane; otherwise, a small random dispersion of normal vector components will lead to large dispersions of the parameter d.

Next, the point clouds are subject to Ransac filtering [42] with a tolerance of 7 mm. This tolerance was set experimentally in the present study by plotting the outliers and ensuring that none of the points evidently lying on the plane was designated as an outlier. The chosen tolerance performed well for most experimental setups. For some setups with long ranges and large incidence angles, the tolerance was manually adapted.

Regular precision estimations in geodetic least squares adjustment processes, such as the Gauss–Markov model, are based on the reference variance, which is then multiplied by the parameters’ covariance matrix, as follows:

(2) $$\hat{\sigma }=\frac{v^{T}Pv}{n-u}$$

where v is the vector of observation residuals, P is the weight matrix, and (n−u) is the number of redundant observations (actual minus necessary number of observations). For the point-cloud-based parameter estimation, the redundancy is far too large to allow for realistic precision estimations.

In our experiments, the fairest estimation was obtained only via multiple repeated scans of the plane followed by computing the covariance matrix of the parameters obtained from the repetitions. We started the testing using triple repetition for the initial plane position and only single scans of the offset positions. We then increased to ten repetitions for the initial position in each setup and, finally, ended at ten repetitions for each setup and offset. This was essential for a reliable precision estimation.

For scanning with Leica MS50, we used only five repetitions since the scanning speed of the total station was much slower, even with a significantly smaller point density. However, the dispersion of the repeated parameters was found to be significantly smaller for Leica scanning, so we assumed that even five repetitions would serve well for fair precision estimations.

### 2.2. Statistical Significance

Using the plane parameters of the initial epoch $p_{0}=\left[\begin{array}{cccc} a_{0} & b_{0} & c_{0} & d_{0} \end{array}\right]$ and their covariance matrix $\Sigma_{p0}$, along with the plane parameters of another measurement epoch $p_{i}=\left[\begin{array}{cccc} a_{i} & b_{i} & c_{i} & d_{i} \end{array}\right]$ and their covariance matrix $\Sigma_{pi}$, we can test if the difference between $p_{0}$ and $p_{i}$ is statistically significant. Since all parameters are of the same type (metric), the overall change of the plane can be defined as the second norm of displacement vector $\Delta_{p}$:

(3) $$\Delta_{p}=p_{i}-p_{0},$$

(4) $$\delta =\sqrt{{\left(a_{i}-a_{0}\right)}^{2}+{\left(b_{i}-b_{0}\right)}^{2}+{\left(c_{i}-c_{0}\right)}^{2}+{\left(d_{i}-d_{0}\right)}^{2}\;}=\|\Delta_{p}\|.$$

The test statistic

(5) $$H=\Delta_{p}{\left(\Sigma_{p0}+\Sigma_{pi}\right)}^{-1}\Delta_{p}^{T},$$

is then distributed through $\chi^{2}$ distribution with four degrees of freedom [43] (p. 334).

For statistical testing of the plane changes, the following hypotheses were set:

- The null hypothesis (H_0_): there is no displacement, δ=0;
- The alternative hypothesis (H_1_): displacement exists, δ≠0.

When the value of H exceeds the critical value of the $\chi^{2}$ test at a given level of significance α for 5% or 1% (corresponding to a confidence level of 95% or 99%), we must reject the null hypothesis (which should never be accepted) and accept the alternative hypothesis with the following statement: “A statistically significant change in the plane occurred”. Otherwise, the alternative hypothesis is rejected.

### 2.3. Experimental Setup

In our research, we investigated how small changes in the plane parameters (see Section 3.2) can be detected by laser scanning. First, we changed only the parameter d by translationally shifting the board based on predefined values without changing the orientation (incidence angle unchanged) of the board (hereafter referred to as a shift test). Second, we tilted the plane at predefined angles to assess the detectable change rate of the normal vector [a, b, c] (hereafter referred to as a tilt test).

It is reasonable to expect that the distance from the scanner to the board and the laser beam’s angle of incidence to the board may affect the detection rate. Other external influences can be omitted by performing tests under laboratory conditions, i.e., with stable meteorological conditions during each test. Some systematic errors in the internal scanners may interfere with the results, but little can be done to mitigate such errors except for extremely careful handling. Later in this paper, we report on the effects of scanner instability that occurred at the beginning of the test scanning.

The experiment consisted of 9 board setups:

- Setups with boards at distances of approximately 5, 10, and 50 m (D5, D10, and D50, respectively);
- Setups with boards at incidence angles of approximately 0°, 40°, and 75° (I1, I2, and I3, respectively).

For the shift test, in each of the nine setups, we shifted the plane by the chosen value in the approximate direction of the plane’s normal. For the tilt test, in each of the nine setups, we tilted the plane by the chosen small angular value around the horizontal axis at the bottom of the board (see Section 2.4.2).

All other interferences and influences were reduced as much as possible.

### 2.4. Instrumentation

#### 2.4.1. Scanning Devices

For this research, two scanning devices were used:

- A terrestrial laser scanner, Riegl VZ-400;
- A robotic total station, Leica MS50.

**Figure 2:**
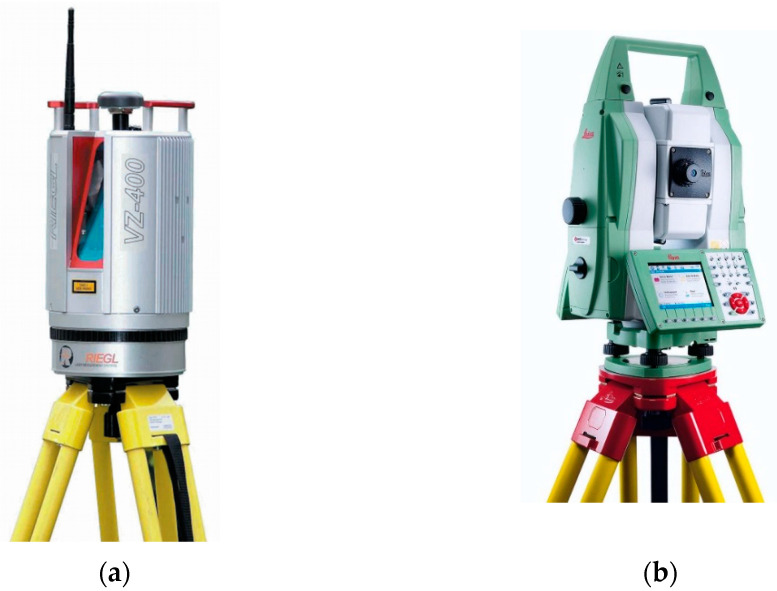
Scanning devices used in the experiments: (**a**) Riegl VZ-400; (**b**) Leica MS50.

The technical specifications of these scanners are listed in Table 1; the scanners themselves are pictured in Figure 2.

We sought to test whether any of the instruments would provide better robustness or stability and whether they would be able to detect smaller changes.

#### 2.4.2. Offset Mechanisms

**Figure 3:**
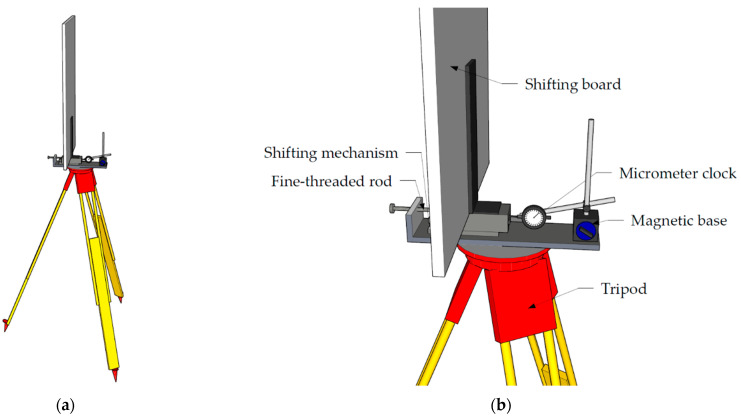
Shift test board setup: (**a**) wooden board on tripod; (**b**) Bahco micrometer with a magnetic base.

For the shift test of the change detection in parameter d, a wooden board of 100 cm × 60 cm was fixed vertically onto a fine-threaded mechanism attached to a surveying tripod (Figure 3). A Bahco micrometer with a magnetic base was also attached to measure the desired shifts.

For setups with different incidence angles, the whole mount was rotated around the heart screw on the tripod table. For these setups, the changes were applied in the direction of the normal vector as desired and not in the direction of the measured distance from the scanner.

**Figure 4:**
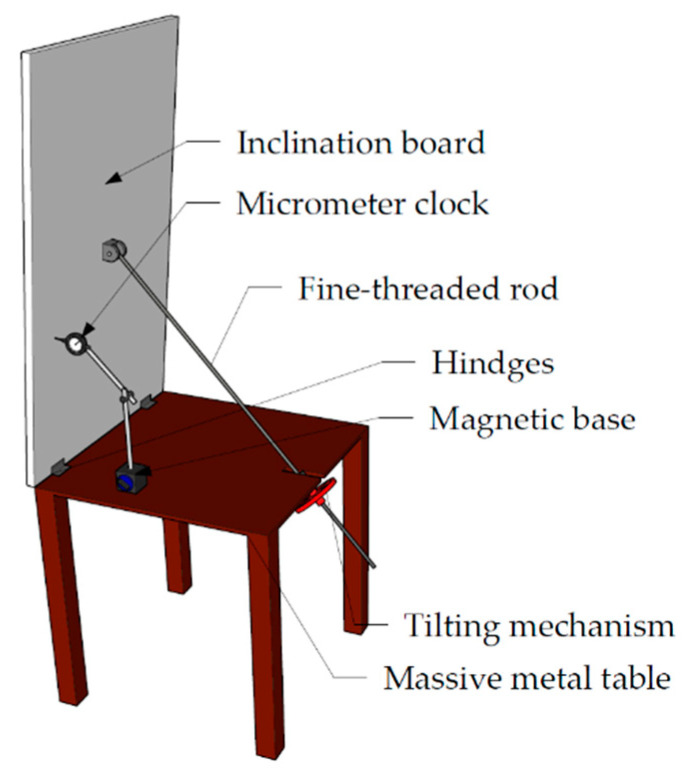
Tilt test board setup.

For the tilt test to detect changes in normal vector inclination, the same wooden board was attached to a massive metal tilting table (Figure 4) with hinges and supported by a fine-threaded rod that was rotatable using a mechanism on the table. This setup allowed us to tilt the plate in a controlled manner. The same Bahco micrometer with a magnetic base was attached to the table to allow precise board declination measurements. The measuring needle was set perpendicular to the board and the radius between the hinges and needle was measured to achieve the desired declination angles.

## 3. Results

### 3.1. Structure of the Results

**Figure 5:**
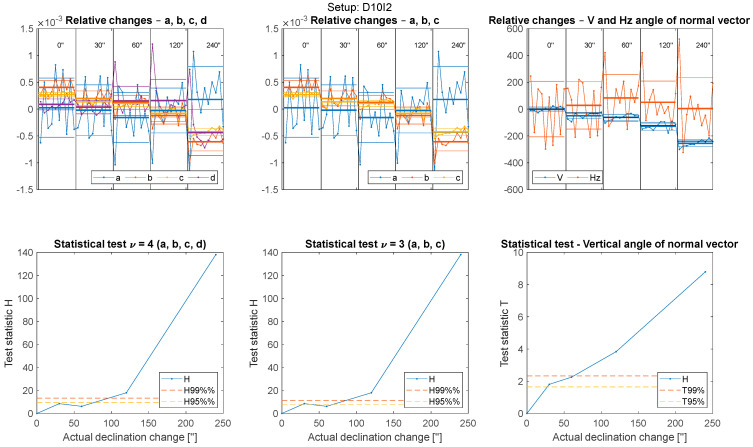
Structure of the results: graphical representation.

To explain the results, we first plotted all values obtained from a single setup (Figure 5). Figure 5 corresponds to the setup at a distance of 10 m and a middle incidence angle (i.e., D10I2).

The upper three frames (Figure 5) present the parameter values subtracted by their means, thereby providing a clearer view of changes in those values.

Vertical black lines designate the physical tilting or shifting of the board. The parameter values of ten repeated measurements are provided in each area.

Each line colour corresponds to one of the plane parameters (a, b, c, and d) in the upper-left frame; only a, b, and c are present in the upper-middle frame. Due to concerns that the significance of the change was caused by unanticipated changes in the parameters (e.g., d in tilt test), we calculated the horizontal direction and vertical angle of the plane’s normal and plotted them in the upper-right frame. For example, when tilting the vertical board (I1), not all parameters should change significantly; in this case, parameter c is expected to change the most. Thus, we must prevent the random dispersion of a and b from causing statistically significant changes to the plane.

Since changes in the normal vertical angle correspond directly to physical changes caused by tilting, the data visualized in the upper-right figure can also be used to assess the accuracy (not only the change significance) of the board declination.

The thick horizontal lines in each area (all colours) correspond to the mean values of the parameters in the area, while the thin horizontal lines indicate the standard deviation. Considering these figures, we sought to determine whether the positions of the thick lines in the first area (0″) were significantly different from their positions in the following areas (30″, 60″, …), while always testing the difference between the initial position (0″) and each of the subsequent positions, and not between successive positions.

Statistical testing was used to produce the lower three frames in Figure 5, where the values of the test statistics are plotted. The lower-left frame presents the results of statistical testing using all four plane parameters. Due to the concerns that d would affect the results of tilting and that a, b, and c would affect the results of shifting, we repeated the same test with parameter *d* omitted (as shown in the lower-middle frame); for the shifting test, a, b, and c were omitted. To produce the lower-right frame, we tested only the changes in the vertical angle. Since only one parameter was tested, normal distribution is used here instead of $\chi^{2}$. The critical values for confidence levels of 95% and 99% are also plotted with dashed horizontal lines to assess the amount of tilting or shifting where statistically significant changes occurred.

In the following section, we focus on the main results separately for the shifting and tilting tests.

### 3.2. Shifting Test

For this test, the board was scanned 10 times with both instruments in the initial position (for the experimental setup, see Figure 3). Then, the board was translated by a chosen value in the direction of the normal vector and scanned 10 times again. The translations were increased until a significant change of the plane was observed.

#### 3.2.1. Translation-Detection Accuracy

**Figure 6:**
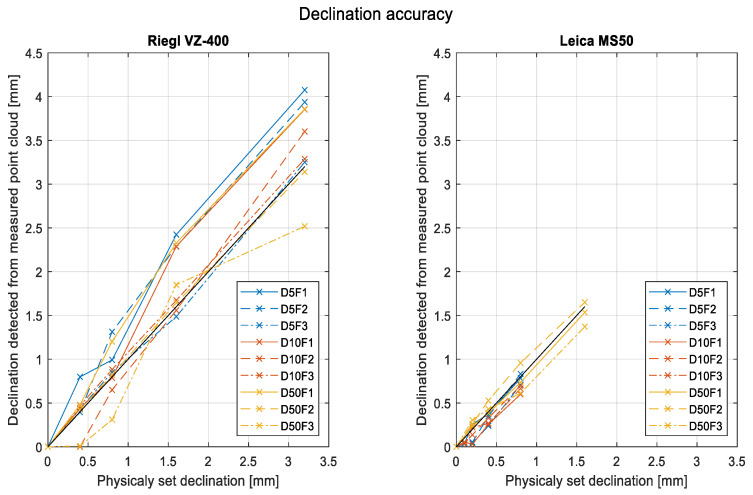
The accuracy of detected shifting declinations.

The accuracy of detected changes in board translation is expressed using the RMSE (Table 2). The errors refer to differences between the physically set offsets or declinations of the board and the changes detected from scanning as changes in the plane parameter d. Differences are plotted in Figure 6.

The numerical values for both instruments and each board setup are listed in Table 2, along with the average RMSEs of all setups. The discussion on these results is in Section 4.2.

#### 3.2.2. Statistical Test

**Figure 7:**
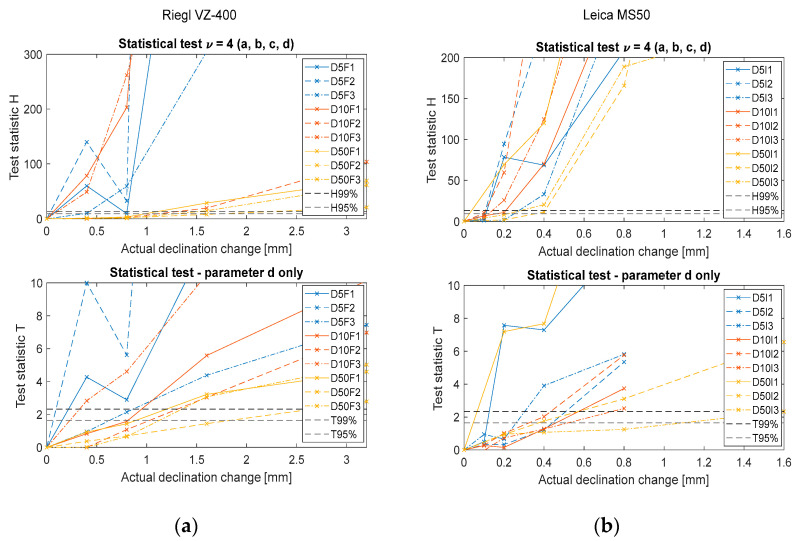
Results of the statistical tests for: (**a**) Riegl VZ-400; (**b**) Leica MS50. Blue lines indicate a distance of 5 m, red lines indicate a distance of 10 m, and yellow lines indicate a distance of 50 m. A full line corresponds to a small incidence angle (I1~0°), a dashed line to an intermediate incidence angle (I2~45°), and a dotted line to large incidence angles (I3~75°). The X axis represents the actual physical declination of the plane and the Y axis represents the values of the test statistics (Equation (5)). The horizontal dashed lines represent the critical values for confidence levels of 95% and 99%.

To remain as concise as possible, we did not render the results for all nine setups and both instruments as done in Figure 5. Instead, we collated only the significant graphs into common figures for all setups (Figure 7).

The left frames present the results for Riegl VZ-400 and the right frames present the same data for Leica MS50.

The upper frames present the results of the four degrees of freedom test (for all four parameters of the plane). Since the board was translated in the direction of the normal vector, we expected that only plane parameter d would change. Therefore, the lower frames show the test statistics only for the significance of changes in parameter d.

In total, we used nine setups at three different distances and with three different incidence angles. Therefore, nine lines are plotted in each frame. The discussion on these results is in Section 4.3.

### 3.3. Tilting Test

For this test, the board was scanned 10 times with both instruments in the initial position. Then, the board was tilted by a chosen value and scanned 10 times again (for the experimental setup, see Figure 4). The declinations increased until a significant change of the plane was observed.

#### 3.3.1. Tilting-Declination Detection Accuracy

**Figure 8:**
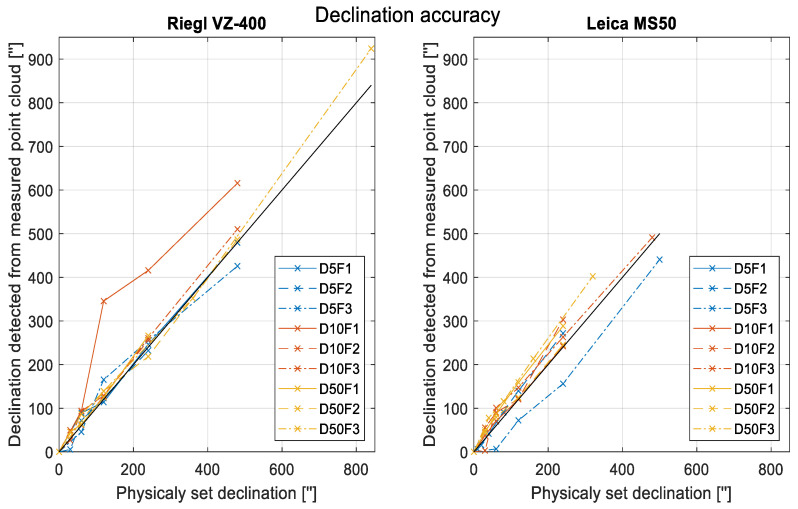
The accuracy of detected tilting declinations.

The accuracy of detected changes in the board inclination is expressed as the RMSE. The errors represent the differences between the physically set tilts or declinations (black line in Figure 8) of the board and the changes detected from the calculated vertical angles of the plane’s normal vector. The differences are plotted in Figure 8.

The numerical values for both instruments and each board setup are listed in Table 3, along with the average RMSEs of all setups. Since the D10I1 graph for Riegl and the D5I3 graph for Leica noticeably deviated from the other graphs, we also computed the average RMSE without these two datasets. The discussion on these results is in Section 4.2.

#### 3.3.2. Statistical Testing

**Figure 9:**
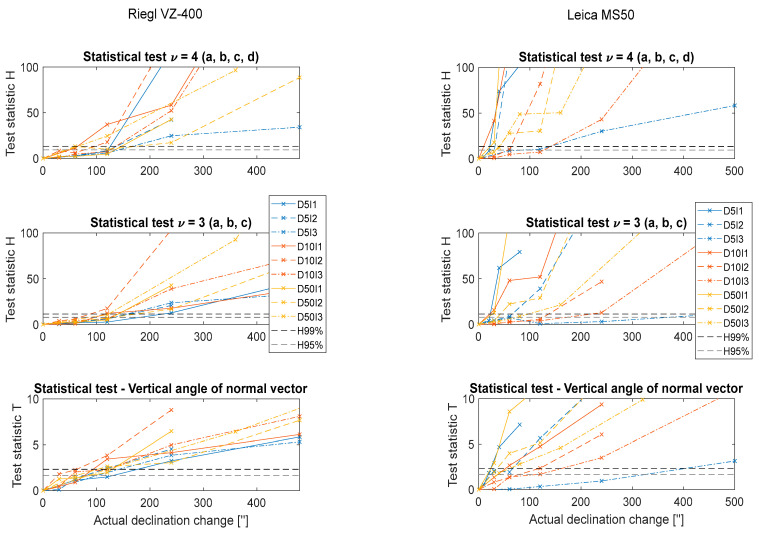
Results of the statistical tests. Blue lines indicate a distance of 5 m, red lines indicate 10 m, and yellow lines indicate 50 m. Full lines correspond to small incidence angles (I1~0°), dashed lines to intermediate incidence angles (I2~45°), and dotted lines to large incidence angles (I3~75°). The X axis represents the actual physical declination of the plane and the Y axis represents the value of the test statistic (Equation (5)). Horizontal dashed lines designate the critical values for 95% and 99% confidence levels.

For the shifting test, we collated only significant graphs into common figures for all setups (Figure 9).

The left three frames present the results for Riegl VZ-400 and the right frames provide the same data for Leica MS50.

The upper frame shows the results of the four degrees of freedom test (for all four parameters), the middle frame shows the results of the three degrees of freedom test (for only a, b, and c since d was not greatly affected by tilting), and the lower frame shows the results from testing the significance of the normal-vector vertical-angle change.

Since we used nine setups at three different distances and three different incidence angles, nine lines are plotted in each frame. The discussion on these results is in Section 4.3.

### 3.4. Single-Parameter Precision

The average dispersion of individual parameters for all setups offered further insight into the precision of the plane parameters obtained by laser scanning. Ten repetitions in each position were performed using Riegl VZ-400 and five repetitions were used for Leica MS50. The numerical values for the precisions of computed parameters for both instruments are listed in Table 4 for the tilt test and in Table 5 for the shift test.

**Figure 10:**
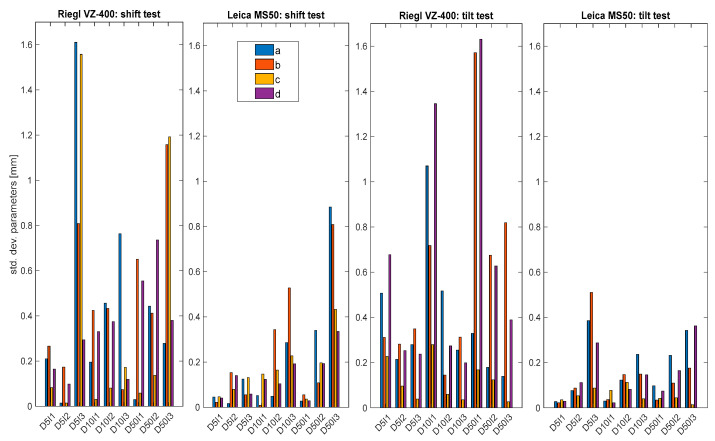
Precisions of the plane parameters.

The precision values are plotted in Figure 10 as bars. Leica MS50 achieved precision approximately two times higher than Riegl VZ-400, even with point density approximately 10 times lower and scanning times 2–3 times longer.

In general, longer distances and larger incidence angles led to slightly lower precision for both instruments, whereas, surprisingly, larger incidence angles provided higher precision for Riegl VZ-400 only in the tilt test.

## 4. Discussion

During the experiments, we acquired results that could be useful in developing guidelines for TLS usage in the field of structural monitoring.

First, the dispersion of the parameters and gross errors suggests that single scanning of an object might be insufficient. Since the scanning itself does not represent the majority of time consumed in the overall process of terrestrial laser scanning, we recommend that the object of interest be scanned multiple times (at least three to five times) in each epoch. In addition to gross-error detection, this method also allows the assessment of the precision of parameters adjusted from point clouds.

**Figure 11:**
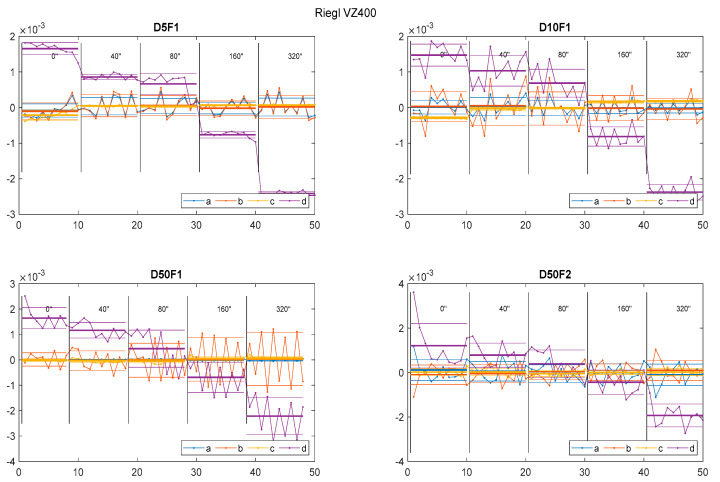
Starting effects of Riegel VZ-400.

Figure 11 shows the start-up effects of Riegl VZ-400, which occurred often during the initial minutes of our test scanning. The dispersion of parameters in the first five to ten repetitions seemed to be significantly larger than those from later in the test. Therefore, we recommend that users warm up their scanners via idle scanning for a few minutes before beginning the process of scanning for real structural monitoring.

### 4.1. Perceived Precision

Some general conclusions can be deduced from Figure 10:

- The total station, despite having lower scanning density, provided precision approximately two times higher than the actual terrestrial laser scanner. For Riegl VZ-400, the average parameter precision was below 1 mm; for Leica MS50, the result was two times better: below 0.5 mm. This result was expected since the nominal specifications of the used instruments outlined the higher precision of the total station.
- Longer distances led to lower precision values for both instruments. Furthermore, in most cases, larger incidence angles led to lower precision.

### 4.2. Perceived Accuracy

Accuracy describes the degree of conformity between the detected deviations and the actual values. For the given dimensions of the board, for both instruments used in the experiment, we can report the following (as shown in Figure 6 and Figure 8 and Table 2 and Table 3):

- For the tilting of the board, the perceived average accuracy for both instruments was between 20″ and 30″. This value corresponds to a tilt value of 0.15 mm per meter. The accuracy decreased with increases in both distance and incidence angles.
- For shifting the board translationally, the perceived average accuracy for Riegl VZ-400 was 0.4 mm and for Leica MS50, 0.1 mm. Significant changes according to distance or incidence angle were not observed.

### 4.3. Statistical Testing-Significance of Changes

Lastly, we present the findings related to our main research question concerning the statistical significance of the detected changes:

- For translational changes of the plane, statistically significant changes were detected when the change was higher than 1.5 mm for the Riegl VZ-400 laser scanner and 0.5 mm for the Leica MS50 total station. However, slightly smaller changes could be detectable at shorter distances and lower incidence angles.
- For the plane-slope changes, statistically significant changes were detected when the change was greater than 150″ for both instruments used in the experiment. This value corresponds to a tilt of 0.7 mm per meter.
- We can see from Figure 7 and Figure 9 that the least steep lines correspond most strongly to large incidence angles and not as significantly to long distances. Therefore, for actual measurements, it is more valuable to ensure smaller incidence angles than shorter distances.
- In real case scenarios, we cannot know whether to expect a shift or tilt of the plane, so we will always use a four degrees of freedom (DOF) test. This might lead to the detection of significant changes too early (in terms of actual displacement). However, we can see from Figure 7 and Figure 9 that the lines from 4DOF tests do not cross the critical value borders much earlier than the lines from 1DOF tests.

## 5. Conclusions

When using TLS to monitor deformations and detect changes, many influencing factors must be considered, such as the stability of the control points, the geometry of the georeferencing, the environmental conditions, and last but not least, the instrumental errors. In this study, we aimed to find out how small changes in the plane parameters can be detected by TLS between two successive measurements, given the same scanner orientation and the same external conditions. With specific instrumentation, we aimed to illustrate the limitations of the TLS in the field of deformation detection on a single primitive. Our work can also be representative of how experts using TLS for deformation monitoring should test their own measuring equipment to find out the limits of their own TLS measuring devices in terms of precision and accuracy.

In addition to the threshold for detectable change, we also compared two scanning devices: a Riegl VZ-400 terrestrial laser scanner and a Leica MS50 robotic total station. In both the plane shift and plane tilt tests, we evaluated the accuracy and precision of the two instruments. Our experiments confirmed the superior accuracy and precision when scanning with the total station, except for tilting detection. The user, however, must also consider the tedious and slow scanning performance of such instruments.

To conclude, the results indicate that through laser scanning of the object and plane adjustment through the point cloud, we are able to detect millimetre shifts of the plane and less than 1 mm per meter in inclination.

Special attention should be paid to possible gross errors when scanning with actual TLS instruments. As well as avoiding the aforementioned start-up effects of TLS, we propose scanning multiple times since the time required for scanning is not as problematic as the benefits of such a procedure.

## Figures and Tables

**Table 1:** Technical specifications of the instruments used in this study.

	Riegl VZ-400	Leica MS50
Temperature operating range	−40 to +40 °C	−20 to +50 °C
Scanner type	hybrid	robotic total station
Vertical declination mechanism	rotating 3-facet mirror	
Horizontal declination mechanism	rotating head	
Field of view	V: +60° to −40°; Hz: 360°	fulldome (except nadir)
Laser	near IR: λ = 1550 nm	visible red: λ = 658 nm
Scanning rate	<122,000 pts/s	1000 pts/s
Range	<600 m	300 m *
Accuracy	Resolution = 1.8″ mm $\sigma_{dist}=$ 5 mm	$\sigma_{angle}=$ 1″ $\sigma_{dist}=$ 1 mm *
Laser beam divergence	3.5 mm/10 m ≅ 72″	8 × 20 mm/50 m ≅ 30″ × 80″

* applies to the 1000 Hz scanning mode used for our experiment.

**Table 2:** The accuracy of detected shifting declinations: RMSE values.

Setup\RMSE [mm]	Riegl VZ-400	Leica MS50
D5I1	0.57	0.04
D5I2	0.51	0.07
D5I3	0.11	0.11
D10I1	0.43	0.14
D10I2	0.29	0.07
D10I3	0.07	0.10
D50I1	0.48	0.04
D50I2	0.04	0.10
D50I3	0.43	0.14
**Average**	**0.38**	**0.10**

**Table 3:** The accuracy of detected tilting declinations: RMSE values.

Setup\RMSE [mm]	Riegl VZ-400	Leica MS50
D5I1	13.6	5.5
D5I2	12.7	18.4
D5I3	33.0	56.0 *
D10I1	142.4 *	5.9
D10I2	11.8	33.2
D10I3	20.4	25.1
D50I1	13.9	31.0
D50I2	17.5	12.7
D50I3	43.4	49.7
**Average**	**52.0**	**31.3**
* Average (gross error excluded)	**22.6**	**26.7**

**Table 4:** Precision of the computed parameters: tilt test.

Riegl VZ-400/Leica MS50	$\sigma_{a}\;[\mathrm{mm}]$	$\sigma_{b}\;[\mathrm{mm}]$	$\sigma_{c}\;[\mathrm{mm}]$	$\sigma_{d}\;[\mathrm{mm}]$	$\sigma_{V}\;[{\;}^{\prime \prime }]$
D5I1	0.51/0.03	0.31/0.02	0.23/0.04	0.68/0.03	48.0/7.4
D5I2	0.21/0.08	0.28/0.09	0.10/0.05	0.25/0.11	34.5/19.2
D5I3	0.28/0.39	0.35/0.51	0.04/0.09	0.24/0.29	52.1/120.4
D10I1	1.07/0.03	0.72/0.04	0.28/0.08	1.34/0.02	59.2/16.6
D10I2	0.52/0.12	0.14/0.15	0.06/0.11	0.27/0.08	22.3/41.4
D10I3	0.26/0.24	0.31/0.15	0.04/0.04	0.20/0.15	43.0/50.4
D50I1	0.33/0.10	1.57/0.04	0.17/0.04	1.63/0.07	35.2/9.1
D50I2	0.18/0.23	0.68/0.11	0.12/0.04	0.63/0.17	43.6/15.4
D50I3	0.14/0.34	0.82/0.18	0.03/0.01	0.39/0.36	38.6/28.7

**Table 5:** Precision of the computed parameters: shift test.

Riegl VZ-400/Leica MS50	$\sigma_{a}\;[\mathrm{mm}]$	$\sigma_{b}\;[\mathrm{mm}]$	$\sigma_{c}\;[\mathrm{mm}]$	$\sigma_{d}\;[\mathrm{mm}]$
D5I1	0.21/0.04	0.27/0.02	0.08/0.05	0.17/0.04
D5I2	0.01/0.02	0.17/0.15	0.01/0.08	0.10/0.14
D5I3	1.61/0.12	0.81/0.06	1.56/0.13	0.29/0.06
D10I1	0.20/0.05	0.42/0.01	0.03/0.15	0.33/0.12
D10I2	0.46/0.05	0.43/0.34	0.08/0.17	0.38/0.10
D10I3	0.76/0.29	0.07/0.53	0.17/0.23	0.12/0.19
D50I1	0.03/0.03	0.65/0.06	0.06/0.04	0.56/0.03
D50I2	0.44/0.34	0.41/0.11	0.14/0.20	0.74/0.19
D50I3	0.28/0.89	1.16/0.81	1.19/0.43	0.38/0.34

## Data Availability

Not applicable.
